# Historical and practical aspects of macular buckle surgery in the treatment of myopic tractional maculopathy: case series and literature review

**DOI:** 10.1186/s40942-024-00578-w

**Published:** 2024-08-28

**Authors:** Francyne Veiga Reis Cyrino, Moisés Moura de Lucena, Letícia de Oliveira Audi, José Afonso Ribeiro Ramos Filho, João Pedro Romero Braga, Thais Marino de Azeredo Bastos, Igor Neves Coelho, Rodrigo Jorge

**Affiliations:** Department of Ophthalmology, Ribeirão Preto Medical School, University of São Paulo, 3900, Bandeirantes Ave, Ribeirão Preto, SP 14049-900 Brazil

## Abstract

**Background:**

Uncorrected myopia is a leading cause of blindness globally, with a rising prevalence in recent decades. Pathological myopia, often seen in individuals with increased axial length (AXL), can result in severe structural changes in the posterior pole, including myopic tractional maculopathy (MTM). MTM arises from tractional forces at the vitreoretinal interface, leading to progressive macular retinoschisis, macular holes, and retinal detachment (RD). This study aims to outline preoperative evaluation and surgical indication criteria for MTM, based on the MTM staging system, and to share our Brazilian experience with three cases of macular buckle (MB) surgery, all with over a year of follow-up.

**Methods:**

We conducted a retrospective analysis of three cases of MTM-associated RD treated with MB surgery, with or without pars plana vitrectomy. Preoperative evaluations included optical coherence tomography (OCT) and ultrasonography (USG) to assess the extent of macular involvement and retinal detachment. Surgical indications were determined based on the MTM staging system. The MB was assembled using customizable and accessible materials. Surgical procedures varied according to the specific needs of each case. An informed consent form regarding the surgical procedure was appropriately obtained for each case. The study was conducted with the proper approval of the institution’s ethics committee.

**Results:**

All three cases demonstrated successful retinal attachment during the mean follow-up of eighteen months. In the first case, combined phacoemulsification, vitrectomy, and MB were performed for MTM with macular hole and RD. The second case required MB and vitrectomy after two failed RD surgeries. In the third case, a macular detachment with an internal lamellar hole was treated with MB alone. These cases highlight the efficacy of MB surgery in managing MTM in highly myopic eyes.

**Conclusions:**

MB surgery is an effective treatment option for MTM-associated RD in highly myopic eyes, providing long-term retinal attachment. Our experience demonstrates that with proper preoperative evaluation and surgical planning, MB can be successfully implemented using accessible materials, offering a viable solution in resource-limited settings. Further studies with larger sample sizes are warranted to validate these findings and refine surgical techniques.

## Introduction

Uncorrected myopia is considered one of the leading causes of blindness worldwide [1], and its prevalence has grown significantly in recent decades [2]. Specifically, in myopic individuals with increased axial length (AXL), structural changes may occur in the posterior pole that characterizes pathological myopia, including posterior staphyloma, myopic macular degeneration, optic neuropathy associated with myopia, and myopic tractional maculopathy (MTM) [3, 4]. The incidence of pathological myopia increases with age but can also occur in younger patients [5]. The impact of myopic maculopathy lies in its frequent occurrence in both eyes, its irreversibility, and its potential to affect individuals of working age [6].

MTM is a specific condition of pathological myopia secondary to tangential and anteroposterior tractional alterations at the vitreoretinal interface, where the retina is unable to adapt to the progressive increase in AXL and ends up undergoing structural changes. Characteristically, it involves a progressive combination of macular retinoschisis, lamellar or full-thickness macular holes, and, ultimately, retinal detachment (RD) [1]. Hence, while antiangiogenic therapy is used to treat neovascular membranes and there is no treatment for atrophic changes, MTM, and its complications require precise surgical interventions, and Macular buckle (MB) surgery, with or without vitrectomy, is one of the surgical techniques options.

In this study, we present the historical aspects of MB, discussing preoperative evaluation and criteria for surgical indication. Hereby we also discuss our experience with MB surgery cases, describing the assembly of a customizable MB using accessible materials.

### Historical context and evolution of the macular buckle

The surgical treatment of RD has undergone revolutionary advancements following the theory developed by Jules Gonin in 1921, which involved surgically blocking tears and breaks in the retina [2]. However, it was soon understood that cases of surgical failure were related to the traction exerted by the vitreous on areas of retinal discontinuity, perpetuating the infiltration of subretinal fluid [3, 4]. In an attempt to alleviate this traction by approximating the underlying choroid to the detached retina, several authors proposed techniques such as subchoroidal injection of plasma, transient indentation with gauze, or even a piece of plastic sutured to the sclera near the treated area [5, 6]. In 1957, Schepens conceived the technique now known as scleral buckling, revolutionizing retinal surgery, and also proposing some adaptations for the treatment of the macular region in cases of retinal detachment associated with macular holes by positioning the buckle beneath the macular region [6].

Over time, other MB techniques were developed by different authors [7–12]. In 1980, Ando [13] created the first solid silicone MB, facilitating its implantation without the need for muscle disinsertion or suturing of the implant to the thinned posterior sclera. However, it presented limitations such as the adjustment of force and interference in imaging exams due to the presence of embedded metal [14]. In 2012, Stirpe et al. developed a new MB that did not contain metal wires and had adjustable sutures [15], while Mateo et al. proposed the coupling of an illuminated probe to facilitate the precise positioning of Ando’s MB beneath the macula [16].

Unfortunately, Ando’s device presents limitations regarding shape, tension adjustment, and posterior suture thus hindering its reproducibility. Hence, certain authors explored alternative methods to tailor their implants, such as utilizing silicone sponges internally coated with stainless steel [17] or employing a titanium stent [18, 19], as described by Parolini et al. (2013). In their report, Parolini et al. detailed three cases where they utilized MB exclusively for macular detachment unrelated to macular holes. Additionally, they introduced a novel L-shaped design of MB devoid of posterior sutures, enhancing its feasibility for surgical implementation [18].

In Brazil, there are no commercially available MBs, so we chose to manufacture one following the descriptions provided by Parolini et al. [18], as we will describe throughout this article.

### Preoperative evaluation, imaging exams in myopic tractional maculopathy, and their role in the surgical indication of macular buckle

Macular buckle surgery requires a comprehensive preoperative ophthalmological assessment and complementary imaging exams to assist in the classification of MTM and surgical planning. Here, we highlight and discuss ocular ultrasonography (USG) and optical coherence tomography (OCT).

### Ocular ultrasonography

The importance of USG in the surgical planning of MB procedures lies in its ability to assess vitreous and retinal conditions, such as the presence of anteroposterior vitreoretinal tractions (VMT) and/or tears, and to locate and estimate the extent of RD. OCT can also be useful for identifying VTM, but standard OCT does not have sufficient width and depth to capture the entire retinal detachment. Sometimes, in eyes with very high myopia, it is challenging to acquire images of the macular holes and, in these cases, examining with the patient using contact lenses can provide better image acquisition. As wide-field OCT is not available in Brazil, USG is very useful in these situations.

USG also aids in selecting the appropriate surgical technique and determining the indication for MB [18, 19]. Moreover, it facilitates the measurement of AXL in cases where optical biometry is unreliable, allows for the accurate calculation of intraocular lens power using the immersion technique to avoid corneal compression [21], assists in identifying structures in cases of media opacity, and ensures accurate intraoperative positioning and postoperative follow-up of the MB. Regarding the anesthetic procedure, USG is essential in evaluating the size of the staphyloma, helping to select the most suitable anesthetic method for highly myopic eyes (retrobulbar block or subtenon anesthesia) to avoid complications such as ocular perforation or intraocular injection of anesthetic in significantly large eyes [22–24].

### Optical coherence tomography

The diagnosis and monitoring of MTM can be challenging due to the atrophic changes associated with pathological myopia. In this context, OCT has emerged as a fundamental diagnostic method for the non-invasive and detailed evaluation of the vitreoretinal interface, retinal layers, the retinal pigment epithelium, and the choroid, allowing for a better understanding and classification of these structures, as described below [25–28].

### Classification and criteria for surgical indication in MTM based on OCT findings

The evaluation of OCT and the correct interpretation of findings are essential steps in surgical indication in MTM. In 2021, Parolini et al. [27–30] introduced a new OCT classification for MTM, which has strong reproducibility between examiners, intending to streamline information sharing and improve understanding of disease progression. [29]. The MTM staging system (MSS) categorizes findings into two types of evolution: perpendicular and tangential. Perpendicular evolution describes the anatomical sequence of predominantly internal or inner retinoschisis (stage 1), predominantly external retinoschisis (stage 2), retinoschisis with macular detachment (stage 3), and complete macular detachment without schisis (stage 4). Tangential evolution, in turn, describes the anatomical sequence of preserved foveal contour (a), internal lamellar macular hole (b), and full-thickness macular hole (c). This classification allows for the combination of evolution types, facilitating disease categorization. The occurrence of external lamellar macular holes is described in the classification as “O”, which can happen at any stage, while the presence of epiretinal abnormalities is indicated as “Plus” [28].

Based on the MSS, a surgical management approach for MTM was proposed. The idea is that comparing MB vitrectomy and pars plana vitrectomy (PPV) alone does not make sense, as each approach has its value in treatment. Early-stage cases warrant observation (stages 1a and 2a), while intervention is reserved for those who experience a progressive decline in visual acuity (stages 1b and 2b). When tangential forces predominate, PPV alone presents good results in stages 1a, with significant epiretinal membrane, and 1b and 1c.

In cases where perpendicular evolution predominates, MB alone has proven effective in stages 2b, 3a, 3b, 4a, and 4b. If epiretinal abnormalities are identified as clinically significant for visual improvement following the MB procedure, rapprochement with PPV remains a viable option. Finally, in cases where perpendicular and tangential forces are present, leading to macular involvement and/or macular or retinal detachment, MB + PPV is indicated (stages 2c, 3c, and 4c). The presence of “plus” alterations may require surgical intervention to improve complaints of metamorphopsia. Table 1 summarizes OCT findings and their implications in surgical indication [30].

**Table 1:** Classification of MTM based on OCT and suggested management of myopic tractional maculopathy [30]

Stage	Characteristics on the OCT	Suggested management
1a	Internal retinoschisis with preserved fovea	Observation
1b	Internal retinoschisis with internal lamellar hole	PPV, if symptomatic
1c	Internal retinoschisis with full-thickness macular hole	PPV
2a	External retinoschisis with preserved fovea	Observation
2b	External retinoschisis with internal lamellar hole	MB, if symptomatic
2c	External retinoschisis with full-thickness macular hole	MB + PPV
3a	Retinoschisis and macular detachment with preserved fovea	MB
3b	Retinoschisis and macular detachment with internal lamellar hole	MB
3c	Retinoschisis and macular detachment with full-thickness macular hole	MB + PPV
4a	Macular detachment with preserved fovea	MB
4b	Macular detachment with internal lamellar hole	MB
4c	Macular detachment with full-thickness macular hole	MB + PPV

**OCT**: optical coherence tomography; **MB**: macular buckle; **PPV**: pars plana vitrectomy

Based on the criteria outlined by Parolini et al. [28–30], we sought to share our experience in this small case series, where all patients underwent MB surgery, with or without PPV, and have been followed up for over a year. Additionally, we will outline the methodology employed for the MB procedure and offer a concise analysis of the results, correlating them with the current literature.

## Methods

This retrospective study analyzed three patients with MTM-associated RD treated with MB surgery, with or without PPV. Preoperative evaluations used OCT and USG to determine macular involvement and the extent of RD. Surgical indications were guided by the MTM staging system, and the MB was assembled using customizable materials. Procedures were tailored to the specific needs of each patient. All participants provided written informed consent. The study received approval from the ethics committee of the Clinical Hospital of the University of São Paulo, Ribeirão Preto, SP, Brazil, and adhered to the principles of the Declaration of Helsinki.

### Cases report

**Fig. 1:**
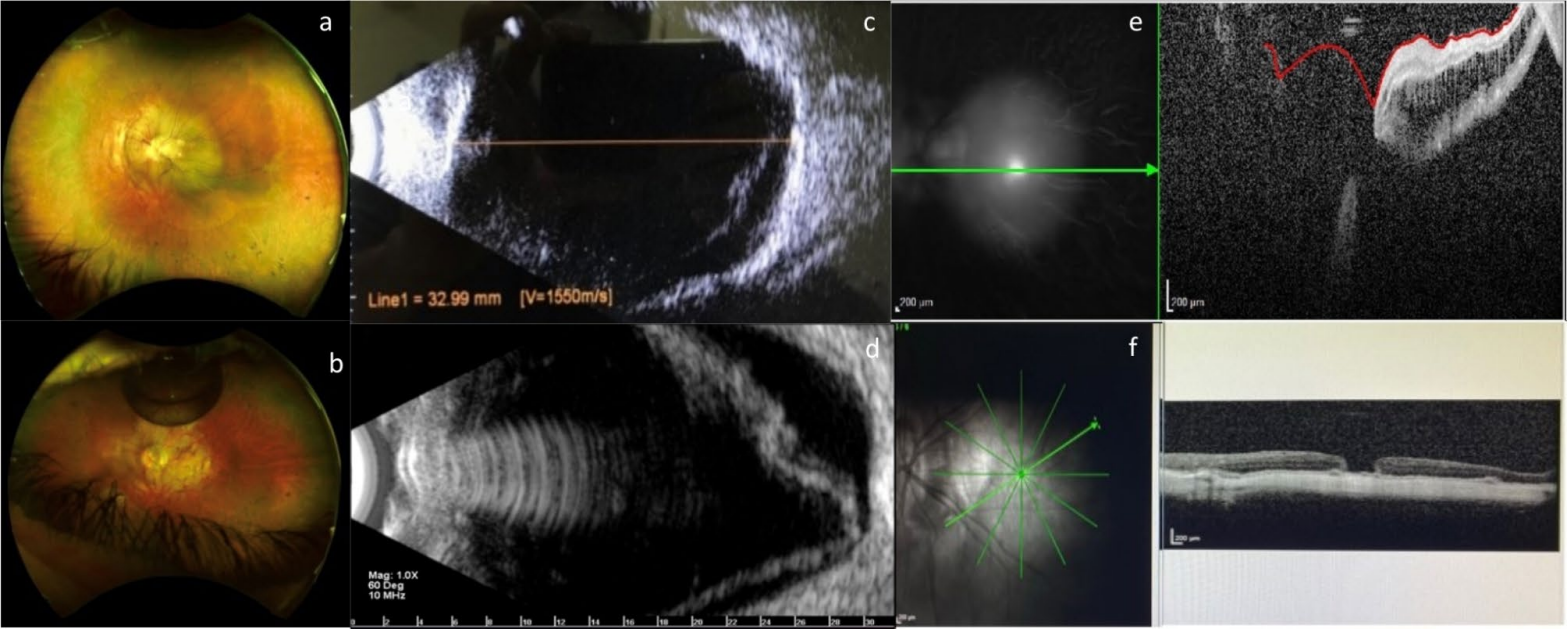
**a**: Color fundus photographs of wide-field preoperative imaging, showing retinal detachment in the posterior pole with a macular hole in the left eye (OS); **b**: Postoperative color fundus photography of the OS with attached retina and a residual gas bubble; **c**: Preoperative USG evidencing retinal detachment and posterior staphyloma; **d**: Intraoperative USG evidencing correct positioning of the buckle flattening the posterior staphyloma; **e**: Preoperative OCT showing a retinal detachment with associated macular hole; **f**: Postoperative OCT showing a reattached retina with a grade 2 macular hole closure exhibiting applied edges (grade 2 closure, Kang et al.’s classification [31])

**Fig. 2:**
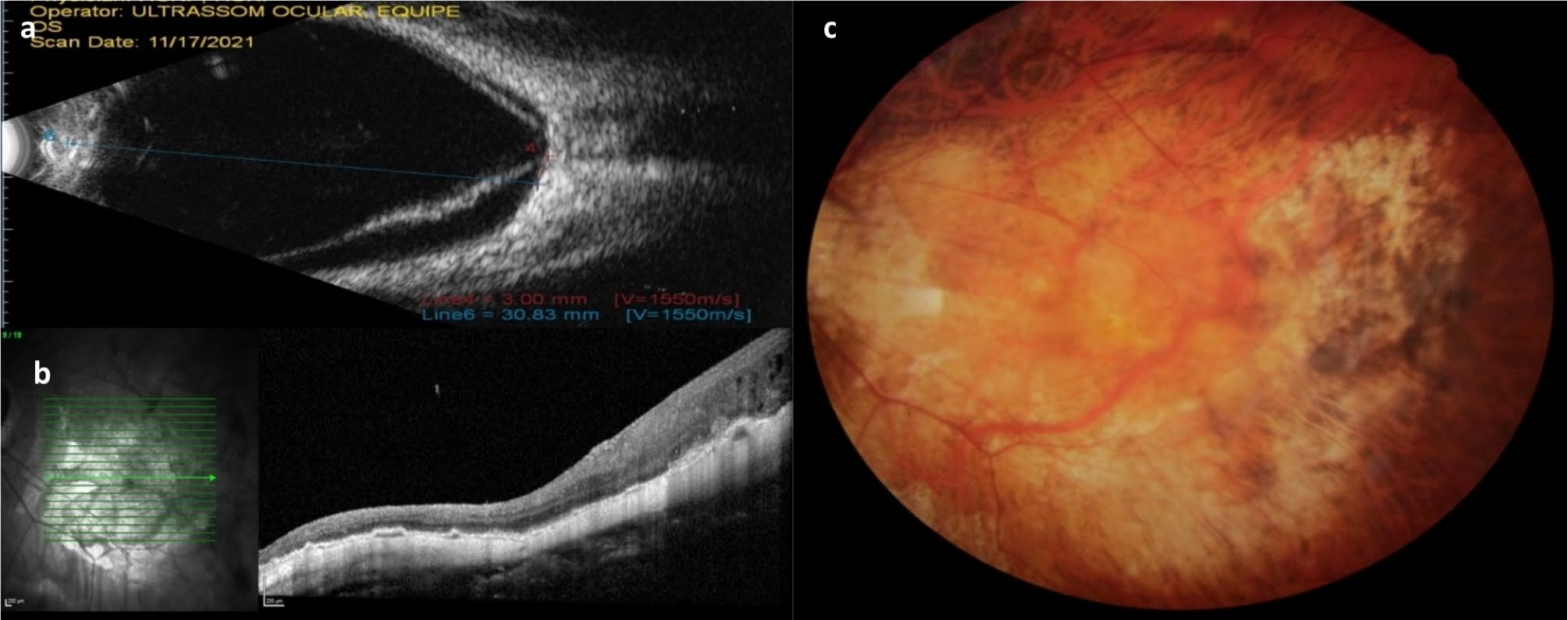
**a**: Ultrasound of the left eye shows retinal detachment; **b**: Postoperative OCT reveals attached retina; **c**: Postoperative color fundus photography of the left eye demonstrates a reattached retina

**Fig. 3:**
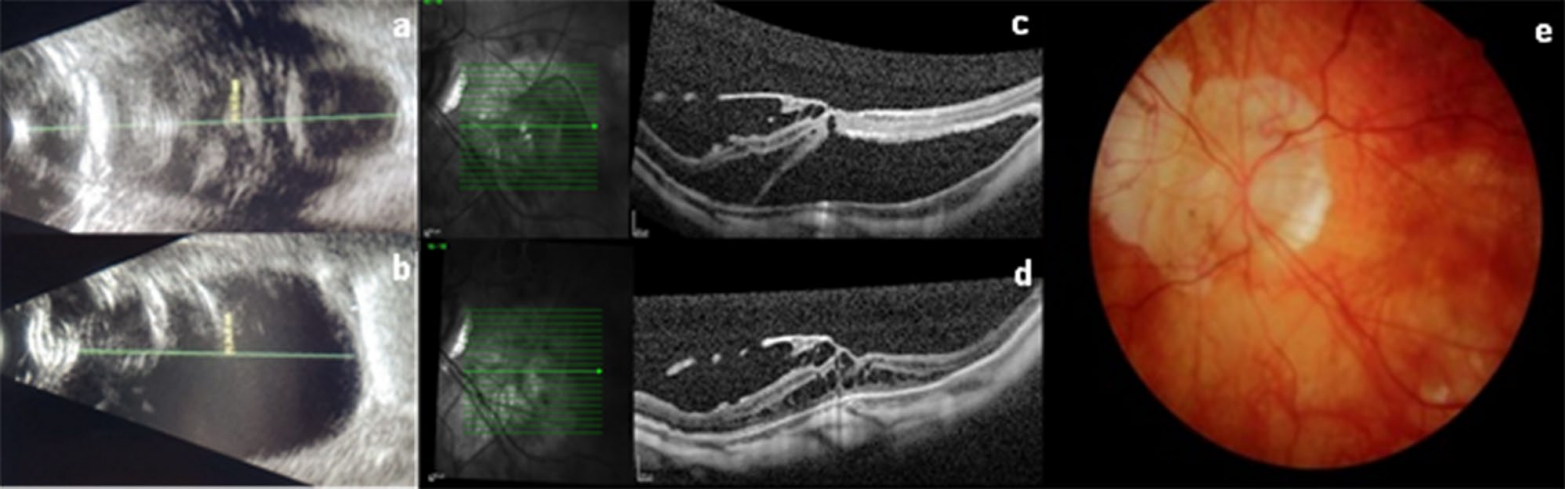
**a**: Preoperative USG showing a large posterior staphyloma with macular detachment (arrow); **b**: Postoperative USG evidencing flattening of the posterior staphyloma due to the positioning of the buckle; **c**: Preoperative OCT showing an internal lamellar hole with macular detachment and nasal macular retinoschisis. Vitreomacular adhesion can also be observed; **d**: Postoperative OCT evidencing flattening of the posterior staphyloma, resolution of the lamellar hole, and macular detachment, as well as reduction of retinoschisis; the vitreomacular adhesion remains stable; **e**: fundus retinography showing attached retina

We describe the surgical management of three cases of highly myopic eyes with MTM, where MB surgery was performed. In cases 1 and 2, RD was associated with a macular hole (MH). In case 2, the indication for MB was due to two previous failures of vitreoretinal surgery (PPV) for the treatment of retinal detachment with a macular hole. In case 3, a macular detachment was associated with an internal lamellar hole. Table 2 summarizes the main findings of each case, and Figs. 1, 2 and 3 illustrate them.

**Table 2:** Demographics and ocular findings before and after the procedure

Case	Sex	Age (years)	Eye	Initial VA	AXL Before surgery	MSS Stage	Previous Surgery	Surgery	Vitreous substitute	Post operative AXL	Final VA
1	Fem	44	OS	CF	32.99 m	4c (RD)	None	Phaco + PPV + MB	C3F8	30.01 mm	20/80
2	Fem	60	OS	CF	30.83 mm	4c (RD)	2 PPV failure for RD	PPV + MB	SO	Not acquired (SO)	20/100
3	Fem	60	OS	CF	32.33 mm	4b	None	MB	None	24.45 mm	20/100

VA: visual acuity; OS: Left eye; CF: counting fingers; AXL: axial length; RD: retinal detachment; Phaco: Phacoemulsification; PPV: pars plana vitrectomy; MB: macular buckle; SO: silicon oil

### Description of implant fabrication and the surgical technique

#### Material

**Fig. 4:**
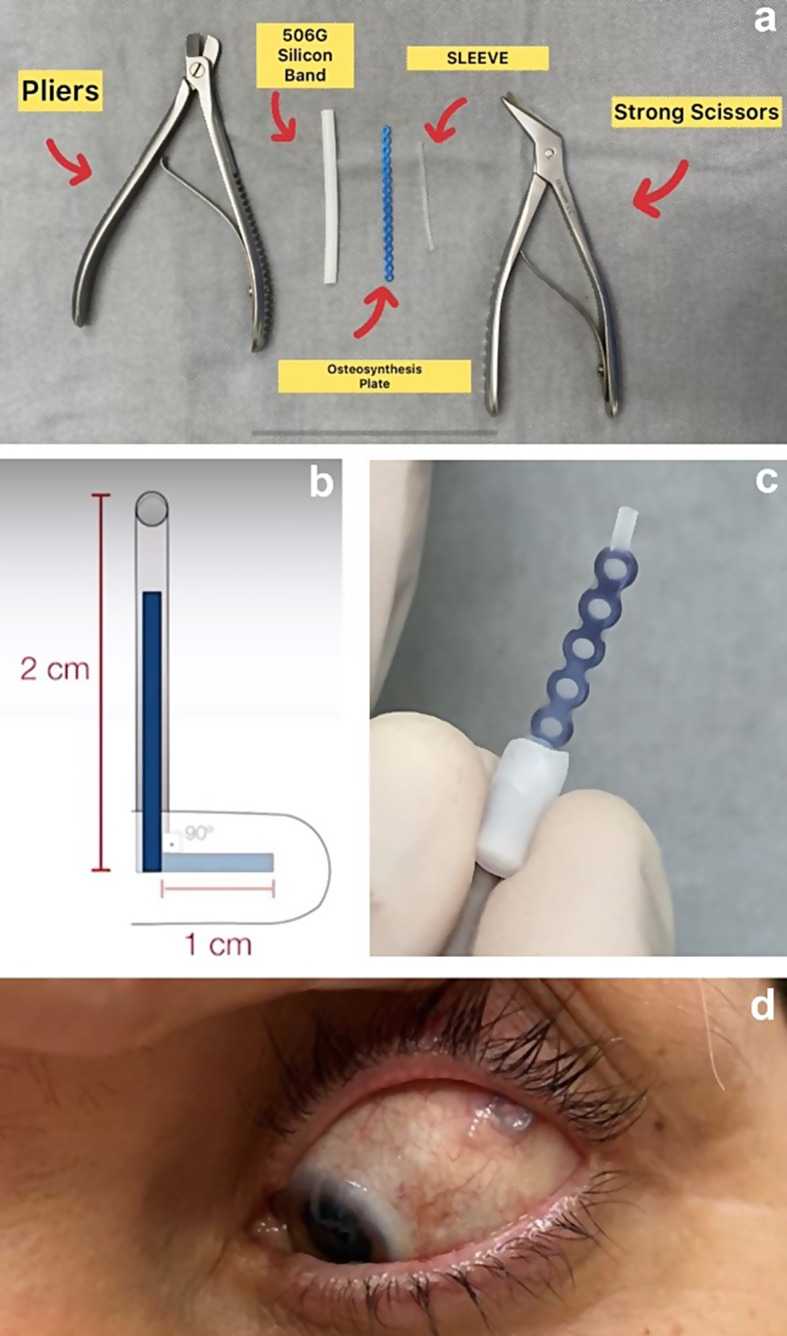
**a**: Material to be used for the fabrication of the macular buckle **b**: Schematic figure of the shape to be molded for the buckle; **c**: MB fabricated in the operating room for the described cases; **d**: Postoperative aspect of the correctly positioned macular buckle; it can be observed under the conjunctiva in the upper temporal quadrant

one 1.5-mm titanium microplate for osteosynthesis containing 8 holes Traumec^®^ (Medical Support, Brazil); one 270 sleeve-type band (Labitician, USA); one 506G oval sponge (Labitician, USA); one 15-degree blade; pliers, and strong scissors (Fig. 4a).

#### Implant fabrication

We used a titanium osteosynthesis plate containing 16 holes, which was cut in half (8 holes) using strong scissors (or pliers), creating the ideal size for our implant. This plate was then inserted into a 270 sleeve-type band (sleeve), covering its entire surface, with the help of Kelly forceps to open the sleeve and facilitate plate insertion, preventing any tearing. Approximately 2.0 mm of the band should be left beyond the plate on the vertical portion to protect the extremity and prevent conjunctival erosion after fixation. The plate is then bent into an “L” shape using pliers, leaving 3 holes horizontally (short arm of the L) and 5 holes vertically (long arm of the L). Next, a tunnel is made in the middle of the linear length of the 506G sponge with a 15-degree blade, ensuring it is longer than the short arm of the titanium plate to cover it, and without letting the tunnel pierce the sponge (to avoid plate exposure). Finally, the short arm of the L-shaped plate is inserted into the 506G sponge through the tunnel, and the 506G sponge should then be cut to cover the short arm of the implant, leaving at least 1.0 mm beyond the implant length to prevent exposure beyond the sponge (Fig. 4a-c).

#### Surgical technique

The initial procedures remain similar, whether isolated MB surgery or combined surgery with vitrectomy is performed. The procedure begins with a temporal peritomy at the limbus of the conjunctiva and Tenon’s capsule from 11 to 4 o’clock. The lateral and superior rectus muscles were isolated using a suture of silk thread 2.0 (Ethicon, Johnson & Johnson, Brazil) to promote eye motility. Before positioning the implant, anterior chamber paracentesis is performed to reduce intraocular pressure (IOP) and minimize pressure changes when positioning the MB. Next, the implant is placed in the upper temporal quadrant, where the shorter arm will be positioned under the macula, and the longer arm should be inserted parallel to the lateral rectus muscle (Fig. 4d). After, a 25-gauge Chandelier optic fiber is positioned at 6 o’clock (Alcon Constellation Vision System, USA) to enable visualization of the fundus.

Subsequently, we confirm the proper positioning of the implant under the macular region using a panoramic visualization system coupled to a microscope (Resight 500^®^, Zeiss) with delicate manipulation of the implant. Once the MB positioning is confirmed, the vertical portion of the device (long arm) is sutured to the sclera using 5.0 Mersilene^®^ suture (Ethicon, Johnson & Johnson, Brazil) with 2 separate stitches. In order to confirm the proper positioning of the MB, we perform preoperative USG, covering the USG probe and cable with a sterile plastic cover, and at the same time, it is possible to measure the comparative AXL.

## Results

As reported above, in two cases, where there was retinal detachment associated to MH, we performed combined MB and PPV surgery (cases 1 and 2), and after positioning the MB, we routinely carried out PPV surgery. In case one, besides PPV and MB, phacoemulsification was carried out, and C3F8 was chosen as a vitreous substitute. In case 2, due to the history of previous PPV and retinal re-detachment with MH, it was decided to use silicone oil as a vitreous substitute in addition to MB. One case presenting an internal lamellar hole (stage 4b) with macular detachment and nasal macular retinoschisis (patient 3) was managed only with MB, despite slight vitreomacular adherence, which was not considered significant.

In the immediate postoperative period of the three cases operated at our service, the patients presented with slight hyperemia, mild pain improved with analgesic (dipyrone), and none showed increased IOP. Patient 3 presented with retinal hemorrhage in the posterior pole in the immediate postoperative period, probably due to the significant reduction of the large preoperative staphyloma after MB implantation. The approach was expectant, and there was complete absorption of the hemorrhage, and progressive reabsorption of the subretinal fluid, leading to the repositioning of the macula throughout the following months, despite a stable vitreomacular adhesion may be seen. In patient 1, during follow-up, the attached retina and grade 2 closure of the macular hole were observed (according to Kang et al.’s classification) [31]. Patient 2 evolved also with retina applied, macular hole closure, and silicon oil. There were no reports of diplopia among the operated patients and/or limitations in ocular mobility.

All three patients (100%) showed visual acuity improvement after surgery, maintaining retina attached and stable vision for more than a year of follow-up. No patient (100%) experienced complications such as conjunctival erosion, displacement/rotation of the MB, endophthalmitis, or anterior chamber reactions throughout the follow-up period.

## Discussion

The use of MB surgery significantly decreased in the 1980s with the advancement of vitrectomy, primarily because of technical difficulties and the lack of related scientific studies at that time [32, 33]. Nonetheless, in highly myopic eyes with posterior staphyloma, PPV can result in surgical failures in 26.7 to 50% of cases due to the inability to alter the axial length of the eye and reduce the anteroposterior forces exerted by the staphyloma [34]. The use of MB in these circumstances can reduce the anteroposterior force, providing positive results. This evidence, combined with the relevant study by Sasoh et al., which demonstrated good results and safety of MB use in the early 2000s, encouraged the resumption of studies and the development of the MB technique [35].

In 2001, Ripandelli et al. [36], compared highly myopic patients with retinal detachment and macular holes undergoing vitrectomy via pars plana (group A) and MB surgery (group B). They observed a surgical success rate of 73.3% in group A and 93.3% in group B, with group B also showing a significant improvement in vision, unlike the vitrectomy group. These results suggested anatomical and functional superiority when MB was used. Similarly, Ando et al., in 2007, reported anatomical success in the MB group in 93.3% of cases after the first surgery and 100% after the second procedure, while only 50% of the cases treated with vitrectomy achieved retinal reattachment in the first procedure, and 86% in the second approach, which was associated with MB [37].

In a literature review, Alkabes and Mateo [32] showed that after MB surgery, the retinal reattachment rate ranged from 81.8 to 100%, while the MH closure rate ranged from 40 to 93.3%. Although persistent MH was identified as a risk factor for retinal re-detachment, eyes with persistent MH that underwent MB did not experience retinal re-detachment. Furthermore, the literature indicates that patients with AXLs greater than 30 mm have a higher risk of early retinal re-detachment after PPV. Several studies have shown statistically significant higher rates of retinal re-detachment after PPV for treating RD associated with MH in patients with AXL > 30 mm [38–40]. For these patients, when undergoing the MB procedure, the retina was reattached in 100% of cases and the MH closure rate ranged from 40 to 100%. Notably, no re-detachment was observed in cases of persistent MH [32]. In our two cases involving RD and MH that underwent combined surgery, both achieved successful outcomes with retinal reattachment and macular hole closure, with no retinal re-detachment observed.

In general, outcomes of both PPV or MB procedures have been shown to be effective in improving retinal anatomy and visual acuity. However, PPV, particularly when combined with internal limiting membrane (ILM) peeling, is associated with a higher incidence of postoperative MH. Due to the lack of randomized studies, it is challenging to determine if MB or PPV is superior for treating progressive macular foveoschisis. Given its progressive nature and potential for RD with MH, surgical intervention should be considered if the schisis progresses or visual acuity decreases. Regular OCT monitoring and early interventions based on physician experience are recommended [32, 41, 42].

Regarding complications, patient 3 experienced retinal hemorrhage following MB surgery, which resolved spontaneously within one month. This patient had a deep staphyloma of the posterior pole, and after MB, the AXL was significantly reduced by 7.9 mm. Despite performing a paracentesis at the beginning of the procedure, no hypotony was observed. We attributed the retinal hemorrhage to the pronounced reduction in AXL. Mateo and colleagues previously described cases where excessive compression of the choroidal vessels could lead to increased local hydrostatic pressure and changes in the RPE, resulting in subretinal fluid and, in some cases, macular atrophy [32, 43]. However, we did not observe any of these complications in patient 3 or the other patients.

Other potential complications reported in various case series include scleral perforation, orbital fat prolapse, improper positioning of the explant, and ocular muscle disinsertion during buckle placement [32]. During the mean follow-up period of eighteen months, no issues such as intraocular pressure changes, strabismus, eye movement restriction, explant displacement, choroidal effusion, choroidal detachment, or posterior pole atrophy were observed.

As demonstrated by Parolini et al., the management of MTM can range from using MB alone to performing combined surgeries. When full-thickness macular holes and macular or retinal detachment are present, a combination of PPV *and* MB is recommended, as each surgical method targets different force vectors affecting MTM [29, 30].

Despite the positive outcomes demonstrated in this report and the literature, MB can present complications. It is essential to carefully evaluate the risk-benefit ratio carefully and reserve its use for cases where it is truly necessary, based on an appropriate classification system. Therefore, we recommend considering MB + PPV surgery as the first choice for highly myopic patients with macular RD associated with MH, given the high rates of retinal re-detachment. In our small case series reported herein, success was achieved with combined surgery in two of our cases and MB alone in one case, proving to be effective in improving anatomical and functional outcomes without the need for additional interventions. None of the patients experienced re-RD with combined surgery or MB alone, which is consistent with the literature.

Finally, it is important to emphasize that the contralateral eye of all three patients continues to be followed up with OCT and fundoscopy. Macular buckling should be considered if any anatomical or visual deterioration occurs, depending on the classification of tractional maculopathy.

## Conclusions

MB has proven to be effective in our small experience, whether alone or conjunction with PPV, in managing MTM. Its indication should consider the pathophysiological mechanism of MTM, which is influenced by tangential and anteroposterior forces, with PPV often needing to be combined in many cases. Decision-making should be based on the patient’s evolution regarding symptoms of decreased vision, anatomical findings on fundoscopy, ocular ultrasound, and based on OCT classification. The postoperative results reported here, and in the literature, have shown good anatomical and functional results, the absence of recurrence of retinal detachment, showing that the macular buckle can contribute to better results in eyes with very long axial lengths.

## Data Availability

No datasets were generated or analysed during the current study.

## Abbreviations

AXL: Axial length
CF: Counting fingers
IOP: Intraocular pressure
MB: Macular buckle
MH: Macular hole
MSS: MTM staging system
MTM: Myopic tractional maculopathy
OCT: Optical coherence tomography
OS: Left eye
PPV: Pars plana vitrectomy
RD: Retinal detachment
SO: Silicon oil
Phaco: Phacoemulsification
USG: Ultrasonography
VA: Visual acuity
